# Topological Optimisation Structure Design for Personalisation of Hydrogel Controlled Drug Delivery System

**DOI:** 10.3390/ma16072687

**Published:** 2023-03-28

**Authors:** Yang Gao, Tan Li, Fanshu Meng, Zhenzhong Hou, Chao Xu, Laixia Yang

**Affiliations:** 1 School of Mechanical Engineering, Xi’an University of Science and Technology, Xi’an 710054, China; 2 State Key Laboratory for Manufacturing Systems Engineering, Xi’an Jiaotong University, Xi’an 710049, China; 3 College of Materials Science and Engineering, Xi’an University of Science and Technology, Xi’an 710054, China

**Keywords:** precision medicine, controlled drug delivery system, hydrogel, topological optimisation

## Abstract

Personalised controlled drug delivery systems (CDDSs) can adjust drug concentration levels according to patient needs, which has enormous research prospects in precision medicine. In this study, the topological optimisation method was utilised in the structural design of a hydrogel CDDS to achieve a parameter-based adjustment of the drug average concentration in the hydrogel. A polyacrylamide/sodium alginate dual-network hydrogel was selected as a drug carrier, and tetracycline hydrochloride was used as a model drug. The topological optimisation model of the hydrogel CDDS was developed. The effects of the mesh size, target concentration, and volume factor on the optimised results were investigated. Hydrogel flow channel structures were obtained, which satisfied the different target concentrations. To verify the rationality of the optimisation model, in vitro drug release experiments were carried out. The results show that the hydrogel CDDS can control drug release within 7 days, and the drug release tends to follow zero-order release behaviour. The adjustable average concentration of tetracycline hydrochloride in hydrogel CDDS is recommended in the range of 20.79 to 31.04 mol/m^3^. This novel method provides a reference for personalised structure design of CDDS in the context of precision medicine.

## 1. Introduction

Genetics-based precision medicine is a “customised” medical model based on a patient’s condition, which has the advantages of convenient diagnosis and accurate medication [1]. Precisely targeted release of drugs is an important part of precision medicine [2]. Consequently, controlled drug delivery systems (CDDSs) have become an area of research focus in the context of precision medicine. CDDSs can be created by integrating medical and industrial science [3,4] in the form of microspheres [5], multilayers [6], nuclear shells [7], micro-cavities [8] and other structures, which have the advantages of precise targeting, low side effects, and high patient compliance [9]. Such CDDSs provide new methods for the long-term treatment and precise control of chronic diseases.

A CDDS can be classified as implantable, oral, injectable, or transdermal in terms of its mode of use [10]. Transdermal CDDSs can avoid the side effects of drugs on the stomach, intestinal tract and the first-pass effect on the liver [11,12]. Hydrogels have been widely used in transdermal CDDSs because of their biocompatibility with the human body and also because they exhibit characteristics similar to natural tissue [13,14]. The hydrogel CDDS can be attached to the wound for drug release, they are beneficial to wound recovery in moist environments while absorbing wound exudates [15]. So hydrogels are used in a variety of applications including wound healing [16], antibacterial activity [17], and pain relief [18]. Xu et al. developed a new nanocomposite hydrogel with a 9 h cumulative drug-release rate of approximately 85% [19]. Yan et al. prepared a hydrogel scaffold for the controlled release of drugs [20]. Lou et al. prepared a drug-loaded hydrogel with controlled drug release within 24 h [21]. Rezanejade et al. studied the drug release behaviour of polyacrylamide/sodium alginate hydrogel-loaded 5-fluorouracil [22]. Parisa et al. prepared chitosan/PVA composite nano-hydrogels to release the tetracycline [23]. Although there have been many studies on the use of hydrogels as carriers for CDDSs, but there are still exist some problems, such as short drug-release time, the burst release at the first stage of delivery, and difficulty in adjusting the drug-release rule.

Currently, in the demand for precision medicine, how to achieve personalised drug release has become the research focus [24]. Changing the microstructure of the hydrogel and chemical modification are the two main approaches. Carafa et al. reduced the drug release rate by loading drugs into a “dual layer” carrier [25]. Li et al. changed the pore size of a hydrogel to control the drug-release rate [26]. Some researchers modified hydrogels by chemical methods, such as changing the combination mode of the hydrogel and drug molecule [27], developing stimulus-responsive hydrogels [28,29,30,31,32]. However, there are few reports on the design of hydrogels’ macro-structure to improve the regularity of drug release.

The topology optimization design method can adjust the structures flexibly by changing the morphology. This method has been used in the aerospace field [33]. Through the topological optimisation design of structure, more achievements have been made in the aspects of heat transfer [34] and weight reduction [35]. In the field of medicine, this method was used for improving the performance of artificial bones and weight reduction [36]. However, there are few reports on the structure design of CDDS based on the diffusion mechanism. In the calculation process of the topology optimisation model, the common methods include the progressive method, the uniform method, and the variable density method [37]. The rational approximation of material properties (RAMP) in the variable density method has the advantages of simple program and high computational efficiency [38].

In this study, the structure of hydrogel CDDS was designed by the topology method to satisfy the needs of on-demand drug delivery. The topological optimisation model of a hydrogel CDDS was developed, and a RAMP interpolation model was chosen to solve it. The minimum difference between the target concentration and average concentration in hydrogel was used as the optimal objective, and the effects of the mesh size, target concentration, and volume factor on optimisation results were investigated. To verify the rationality of the model, the CDDS with topology structural mould were prepared by 3D printing, and the hydrogel CDDS was tested in vitro. This study proposes a novel way to personalise the structural design of CDDSs, and provides the theoretical support for the on-demand drug delivery.

## 2. Materials and Methods

### 2.1. Materials Introduction

A polyacrylamide-sodium alginate dual-network hydrogel with well controlled drug-release properties was selected as the drug carrier [39]. Acrylamide (C_3_H_5_NO, Lot No. 20220401), sodium alginate ((C_6_H_7_NaO_6_)_n_, Lot No. 20210901), N, N-methylene-bis-acrylamide (C_7_H_10_N_2_O_2_, Lot No. 20210115), ammonium persulfate ((NH_4_)_2_S_2_O_8_, Lot No. 20211023) and calcium chloride anhydrous (CaCl_2_, Lot No. 20210710) were all purchased from Sinopharm Chemical Reagent Co., Ltd., Shanghai, China.

For its broad-spectrum antibiotic in the pharmaceutical field, the tetracycline hydrochloride (C_22_H_24_N_2_O_8_·HCl, Lot No. G2116343, Shanghai Aladdin Biochemical Technology Co., Ltd., Shanghai, China) was used as a model drug [40]. The molecular weight is 480.9 g/mol and its saturation concentration in water is 41.59 mol/m^3^.

### 2.2. Forming and Preparation Methods

When the dual-network hydrogel was prepared, acrylamide and sodium alginate monomers were added to water first. N, N-methylene-bis-acrylamide was then added as a cross-linking agent, and ammonium persulfate was used as the catalyst. The mixed solutions were stirred, and the bubbles generated by the stirring were treated in a vacuum chamber. Furthermore, the uniformly mixed solutions were poured into a mould. The ambient temperature was adjusted to 60 °C to allow the mixed solutions to solidify into a hydrogel. Finally, the hydrogel was immersed in a CaCl_2_ solution and swelled until it reached equilibrium.

Diffusion studies were performed using a side-by-side glass diffusion cells based on concentration gradient method [41,42,43] where the temperature 37 ± 0.5 °C was controlled by a water bath (LICHEN, DF-101S). A magnetic stir bar was placed in each half-cell for continued agitation. Between the two half-cells, a pre-equilibrated hydrogel membrane was securely placed and protected from the atmosphere to prevent evaporation of the solvent from the membrane. 5 mL of the supersaturated drug solution and 5 mL of the solvent were added to the donor half-cell and the receptor half-cell respectively. Until a stable concentration gradient was formed in the hydrogel, the contents of the receptor cell were moved and replaced with fresh solvent at regular intervals. The concentration of each sample taken from the receptor half-cell could be determined by a high-performance liquid chromatograph (HPLC, SPD10A, Shimadzu, Tokyo, Japan) with a wavelength of 280 nm. Furthermore, the flux of tetracycline hydrochloride through the hydrogel membrane was calculated. According to Fick’s law of diffusion, the average diffusion coefficient of tetracycline hydrochloride through hydrogel was confirmed.

3D printing is appropriate for preparing complex structures [44], especially in the formation of topology optimised structures. Therefore, in this study, liquid crystal display (Anycubic Photon Mono SE) was applied to prepare the mould of topology optimised flow channels. During the printing process, the thickness of each layer was 0.04 mm, the normal exposure time was 15 s, the light off time was 10 s, the bottom exposure time was 50 s, and the number of bottom layers was 5.

In the in vitro experiment, the prepared hydrogel CDDS was put into 200 mL of normal saline at a temperature of 37 ± 0.5 °C. The solution was taken out at intervals and determined by a high-performance liquid chromatograph (HPLC, SPD10A, Shimadzu, Tokyo, Japan) with a wavelength of 280 nm. After each test, it was replaced by a new saline solution. The cumulative drug release was measured and the curve with the drug release time was plotted.

### 2.3. Optimisation Model for Hydrogel CDDS

**Figure 1:**
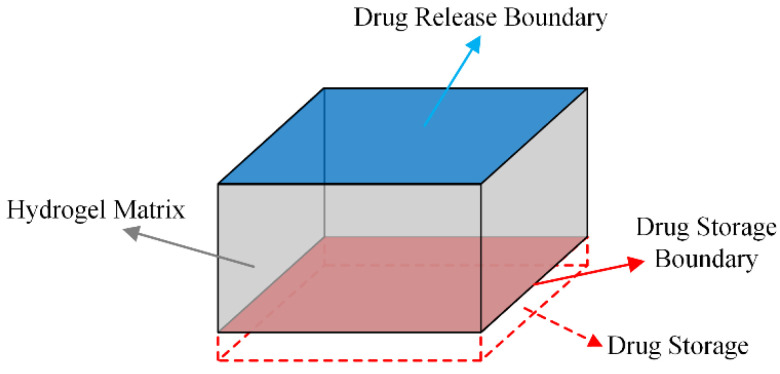
The structural schematic of the hydrogel CDDS before optimisation.

The structural schematic of the hydrogel CDDS before optimisation is shown in Figure 1. The dotted line represents the drug reservoir used to store sufficient drugs. The red area represents the drug-reservoir boundary, and the grey solid area represents the hydrogel used as a channel to deliver the drugs. The blue area represents the drug-release boundary for tetracycline hydrochloride release.

In this study, the hydrogel CDDS was topology-optimised under the premise of swelling equilibrium. Consequently, the following assumptions were made for the mass transfer process of drugs in hydrogels:

(1) Because the polyacrylamide-sodium alginate dual-network hydrogel degrades insignificantly when stored in water for 6 months [45], the effect of the degradative property of the hydrogel on drug release was ignored. During optimisation, the mechanism of drug release was diffusion, and the diffusion coefficient of the drug in the hydrogel was constant.
(2) In order to meet the clinical conditions as much as possible, the parameters in the model were all values at a temperature of 37 °C.
(3) The drug was only released from the top surface.

Based on the above assumptions, the drug release followed the law of Fick’s diffusion. The boundary and initial conditions of the drug release model were set as follows:

(1) The drug-reservoir boundary is the red boundary shown in Figure 1, during the drug delivery, its concentration was 41.59 mol/m^3^, which is the saturation concentration of tetracycline hydrochloride.
(2) The no-flux boundary is the grey boundary shown in Figure 1, the tetracycline hydrochloride was prevented diffuse from this boundary.
(3) The drug diffusion boundary is the blue boundary shown in Figure 1, the tetracycline hydrochloride diffuses into the external environment through this boundary during drug delivery. For the reason that the external environment simulates the state of body fluid circulation, if any drug diffuses through this boundary, it is removed by the circulation of body fluids. So that the concentration of this drug diffusion boundary was always 0 mol/m^3^.
(4) According to the method in Section 2.2, the diffusion coefficient of tetracycline hydrochloride in polyacrylamide-sodium alginate hydrogel was 1.8 × 10^−11^ m^2^/s, and diffusion coefficient of tetracycline hydrochloride in water was 7.42 × 10^−10^ m^2^/s.

The variable density method was used to optimise the topology structure, the real material was replaced with the density value in the design process. Any value within the range of 0 to 1 can be used as the density value, 0 represents the solid structure with the hydrogel, and 1 represents the flow channel without the hydrogel. During optimisation, the hydrogel area was divided into meshes, and the density value of the mesh was regarded as a design variable for optimising the hydrogel structure. The mathematical model of optimisation can be expressed as follows:

$$\mathrm{Find}:\;x=(x_{1},\;x_{2},\;x_{3},\;\ldots ,\;x_{i},\;\ldots ,\;x_{n})\in [0,1]$$

(1) $$\begin{array}{c} \int x\;d\Omega \leq q_{v}V_{0} \end{array}\;$$

0 < *x_min_* ≤ *x_i_* ≤ 1                                                                          

where *x* is the density value of the mesh, *q_v_* is the volume factor used to limit the proportion of the flow channel in the hydrogel, and *V*_0_ is the initial volume of the hydrogel.

Under constant boundary and initial conditions, the cumulative release of CDDS can be determined by the average concentration in the hydrogel. So that the minimization of the difference between the drug target concentration and the drug average concentration in hydrogel was used as the optimal objective:

(2) $$\begin{array}{c} min:f(x)=\left|C_{obj}-C_{avg}\right| \end{array}$$

(3) $$\begin{array}{c} C_{avg}=\frac{\int C\left(x\right)dx}{V_{0}} \end{array}\;$$

where *C*(*x*) is the concentration of the design variables, *C_obj_* is the pre-specified target concentration of the hydrogel, *C_avg_* is the average concentration of the hydrogel in the current structure, and *f*(*x*) is the absolute value of the difference between the average concentration in the hydrogel and the target concentration.

### 2.4. Key Methods and Parameters for Solving the Optimisation Model

COMSOL Multiphysics was used to design topological flow channels in the hydrogel CDDS. Based on the variable density method, the hydrogel was identified as a material which density varies continuously from 0 to 1. Intermediate-density values were created during the optimisation process. A penalty coefficient was introduced to penalise the intermediate-density values, making the diffusion coefficient tend towards the binomial distribution as much as possible. The RAMP model was selected to develop a function representing the density values and diffusion coefficients, it was expressed as follows:

(4) $$\begin{array}{c} D(x_{i})=\left(D_{max}-D_{min}\right)\frac{x_{i}}{1+q\left(1-x_{i}\right)}+D_{min} \end{array}\;$$

where *D*(*x_i_*) is the diffusion coefficient of the corresponding mesh, *D_min_* is the diffusion coefficient of the drug in the hydrogel, and *D_max_* is the diffusion coefficient of the drug in the flow channels. Ideally, when *x_i_* = 0, the diffusion coefficient of this mesh is the diffusion coefficient of the drug in the hydrogel, and when *x_i_* = 1, the diffusion coefficient of this mesh is equal to the diffusion coefficient of the drug in saline. *q* is the penalty coefficient.

The intermediate-density values were penalised, which effectively reduces their number. However, the distribution of density values can also generate numerical instabilities, such as tessellation. To avoid this problem, the density filter method was used to handle numerical instabilities, which can be achieved by coupling the Helmholtz-type partial differential equation:

(5) $$\begin{array}{c} x_{f}=x+R_{min}^{2}\nabla^{2}x_{f} \end{array}\;$$

where *R_min_* is the filtering radius, *x_f_* is the filtered density, and ∇ is the Hamiltonian counter.

Numerical instability can be effectively avoided by density filtering. However, intermediate-density values were generated. To obtain a topology with a clear boundary, the intermediate values obtained after filtering were projected using a projection function:

(6) $$\begin{array}{c} x_{p}=\frac{\tanh\left(\beta \left(x_{f}-x_{\beta }\right)\right)+\tanh\left(\beta x_{\beta }\right)}{\tanh\left(\beta \left(1-x_{\beta }\right)\right)+\tanh\left(\beta x_{\beta }\right)} \end{array}\;$$

where *β* is the slope of projection, which can be used to control the steepness of the projection, *x_β_* is the threshold value of projection, and *x_p_* is the density value obtained after projection.

During the optimisation process, the method of moving asymptotes (MMA) was used as the iterative algorithm, the convergence condition was set as follows:

(7) $$\begin{array}{c} max:\left|x_{i}^{k}-x_{i}^{k-1}\right|\leq 10^{-3},\;\;\;k\in \left(0,1000\right] \end{array}$$

where *k* is the number of iterations, $x_{i}^{k}$ is the density value when the number of iterations is *k*, $x_{i}^{k-1}$ is the density value when the number of iterations is *k* − 1. Convergence was completed when the tolerance of convergence was reduced to 10^−3^, and the maximum number of iterations is 1000.

**Figure 2:**
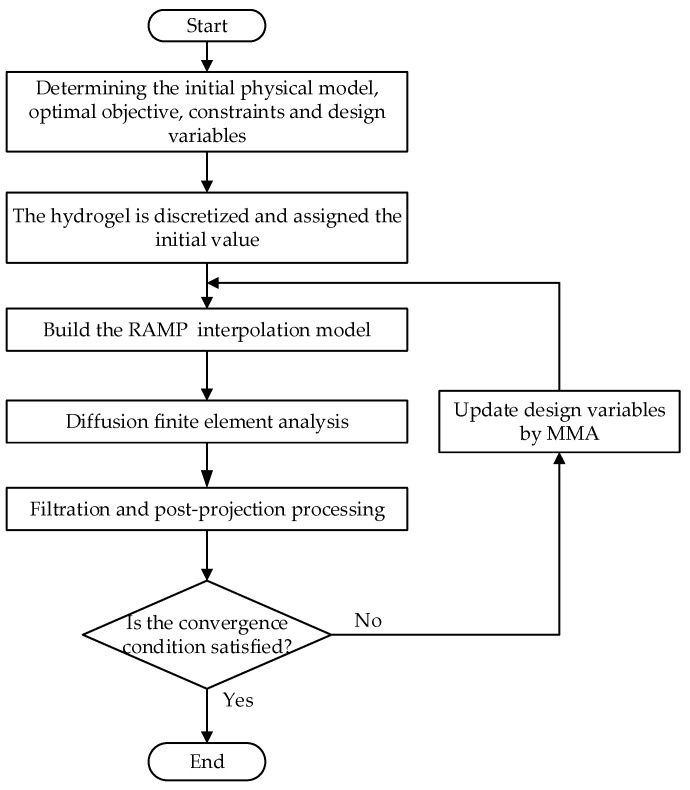
Topological optimisation design flow chart.

Figure 2 shows the specific hydrogel CDDS optimisation process.

## 3. Results and Discussion

The purpose of topological optimisation is to achieve parametric control of the released drug in the hydrogel CDDS by changing the average concentration in the hydrogel. Consequently, the range of target concentrations in the hydrogel was first determined.

**Figure 3:**
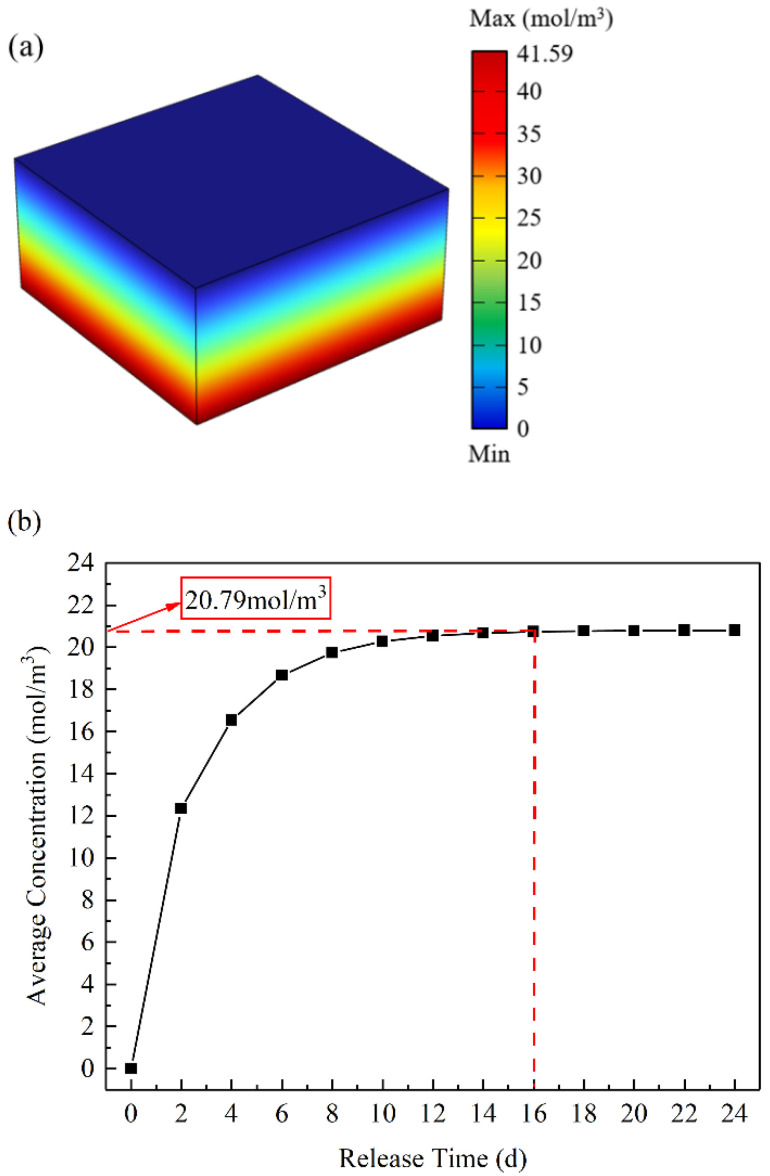
Numerical simulation results of the CDDS without structure optimisation: (**a**) Drug concentration in the hydrogel. (**b**) The curve of average hydrogel concentration with release time.

A drug-release simulation was conducted on the initial model of the CDDS without any flow channels in the hydrogel, the results of which are shown in Figure 3a. A concentration gradient was formed in the hydrogel, which drove the CDDS to release tetracycline hydrochloride to the outside steadily and continuously. Figure 3b shows the relationship between the average concentration in the hydrogel and the time of drug release. It is evident that the drug diffuses rapidly at the beginning of release, the average concentration in hydrogel tending a constant value of 20.79 mol/m^3^ after 16 d, and a stable concentration gradient in the hydrogel was formed. This value is the average concentration that can be formed in the hydrogel without flow-channel. The saturation concentration of tetracycline hydrochloride in saline at 37 °C is 41.59 mol/m^3^. In this study, the target concentration for topological optimisation of the hydrogel CDDS was in the range of 20.79 to 41.59 mol/m^3^.

### 3.1. Effect of Mesh Size on Optimisation Results

During the optimisation of the hydrogel CDDS, the density value of each mesh varied as the optimisation progressed, the density value distribution of the mesh determining the structure of the flow channel. In the case of a consistent mesh shape, the optimisation results are dependent on the mesh size [46].

**Figure 4:**
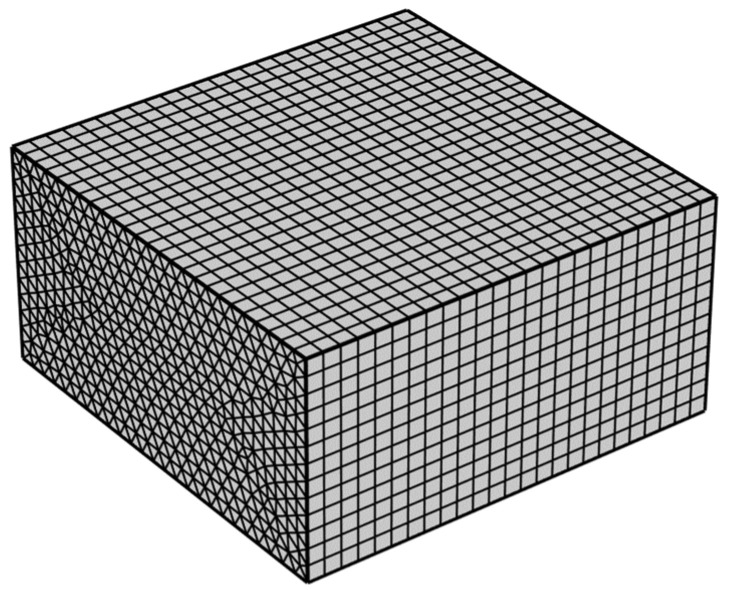
Meshes of the hydrogel CDDS.

The model was divided by trigonal column meshes, as shown in Figure 4. The filter radius was 0.2 mm, the penalty coefficient was 8, and the projection slope was 10. With a hydrogel target concentration of 28 mol/m^3^ and a volume factor of 0.1, to study the effect of the mesh size on the optimisation results, mesh sizes of 0.08, 0.1, 0.2, 0.3, 0.4, and 0.5 mm were used, respectively.

**Figure 5:**
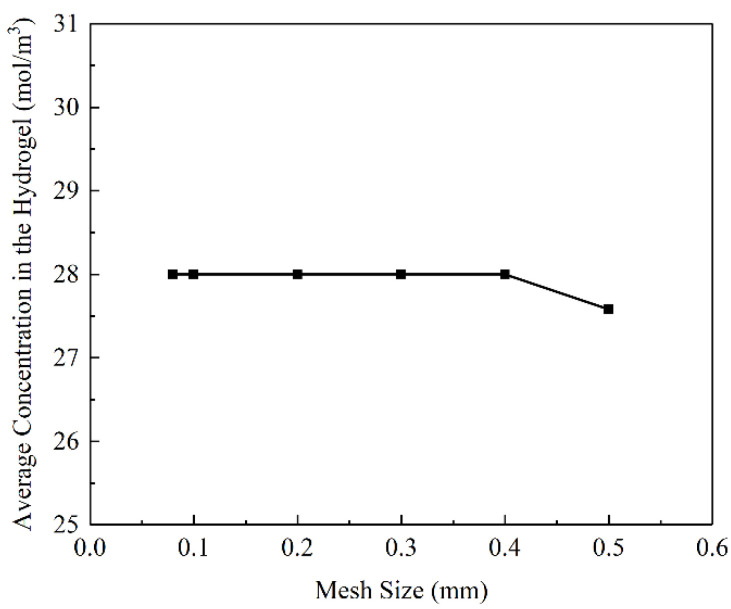
Relationship between the mesh size and average concentration in the hydrogel.

As shown in Figure 5, the average concentration in the hydrogel changes with the mesh size. A target concentration of 28 mol/m^3^ can be achieved when the mesh size is less than 0.5 mm.

**Figure 6:**
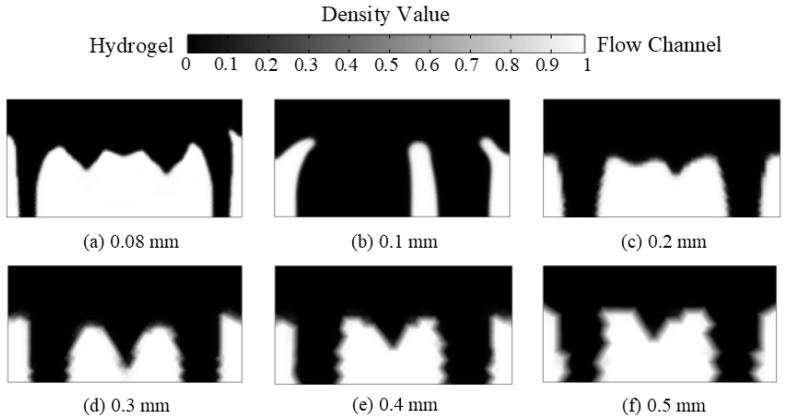
Density value distributions of cross-sections using different mesh sizes.

Figure 6 shows the density value distribution of the CDDS with different mesh sizes. As the mesh size decreases, the outline of the flow channel becomes clearer, and the jagged phenomenon gradually disappears. The flow channel boundary is clear and smooth at a mesh size of 0.1 and 0.08 mm.

**Figure 7:**
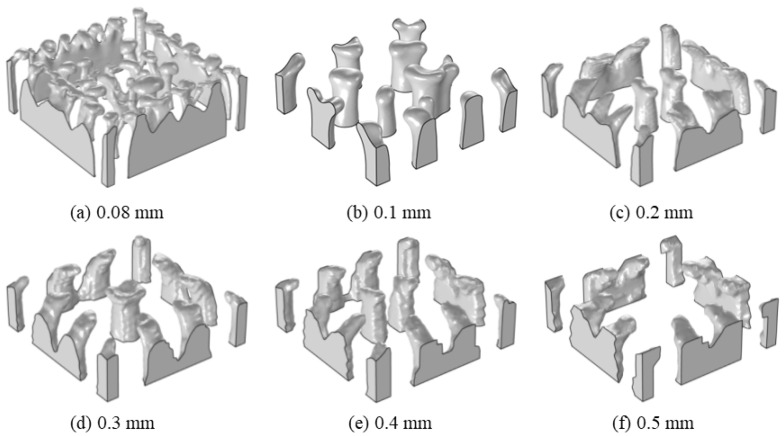
Flow channel structures using different mesh sizes.

Figure 7 shows the optimised structures of flow channels using different mesh sizes. Follow the changes in mesh sizes; the flow channel structures are different. With mesh sizes changed from 0.2 to 0.5 mm, the flow channel structures have the feature of medial extension. When the mesh size is 0.08 mm, there are many small branches in the flow channel.

Overstretch and small branches both result in manufacturing difficulties. At a mesh of 0.1 mm, the surface of channel structures is smooth and without small branches, which is beneficial for the hydrogel demoulding.

Based on the above discussions, at a mesh size of 0.1 mm, the average concentration in hydrogel CDDS can be achieved, and the shape of flow channels is smooth and contributes to manufacture. A mesh size of 0.1 mm was chosen for subsequent analysis.

### 3.2. Effect of Target Concentration on Optimisation Results

The effects of different target concentrations on the optimisation of the flow channel structure and the average concentration in hydrogel were studied. The target concentrations were changed to 24, 26, 28, 30, and 32 mol/m^3^, with a mesh size of 0.1 mm and a volume factor of 0.1. 

**Figure 8:**
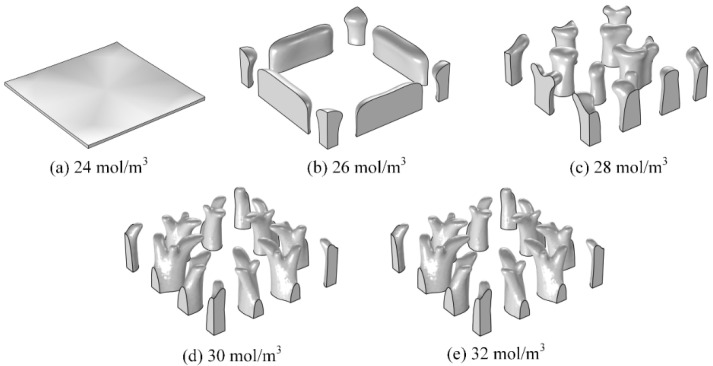
Flow channel structures for different target concentrations.

Figure 8 shows the optimised structures of the flow channels for different target concentrations. The volume of flow channels increased with target concentration. If the target concentration is small, overmuch flow channels are unnecessary (Figure 8a,b). The structures of the flow channels are highly consistent when the target concentrations are 30 and 32 mol/m^3^. If the target concentration keeps increasing, the optimised structures of the flow channels will remain unchanged. That means 30 mol/m^3^ maybe the maximum concentration that can be adjusted with the current parameters.

**Figure 9:**
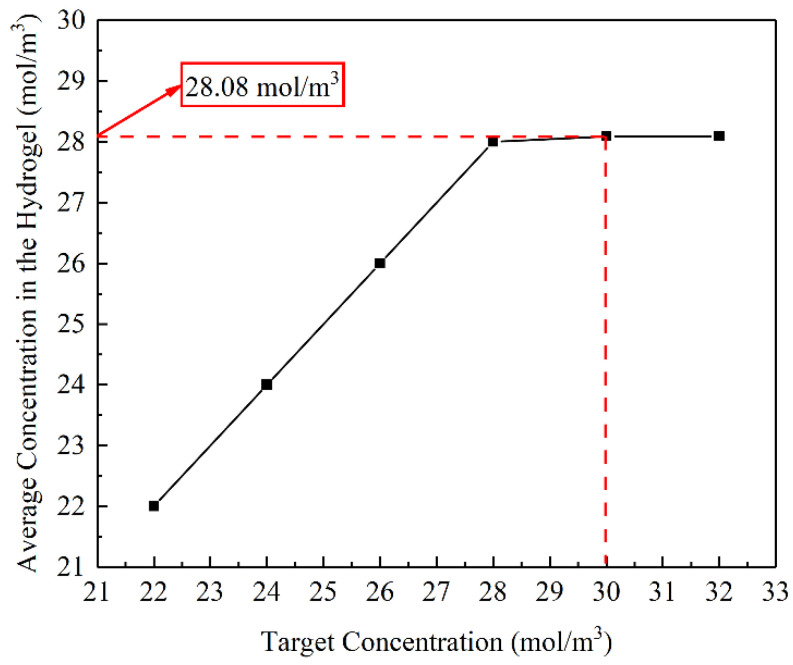
Relationship between target concentration and the average concentration in the hydrogel.

Figure 9 shows the relationship between the different target concentrations and the average concentrations in the hydrogel. When the target concentrations are less than 28.08 mol/m^3^, the average concentration in the hydrogel can reach the set value. Whereas, when the target concentrations exceed 28.08 mol/m^3^, the average concentration in the hydrogel is maintained at 28.08 mol/m^3^. So that the maximum concentration in the hydrogel which can be adjusted is 28.08 mol/m^3^. This is consistent with the above conclusion.

**Figure 10:**
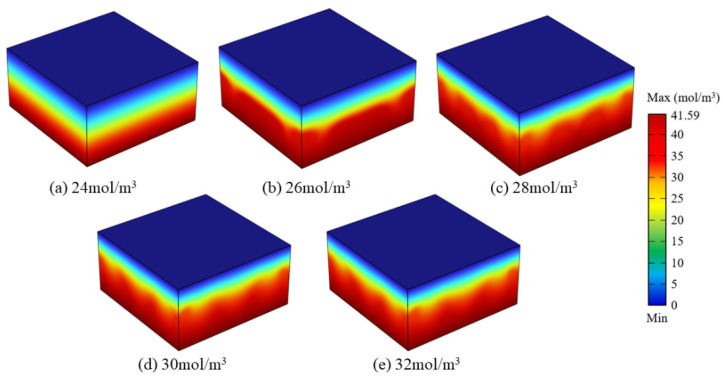
Concentration distributions for different target concentrations.

Figure 10 shows the concentration distributions of the optimised hydrogels. Compared with Figure 3a, the concentration distribution distinctly changes after optimisation. When the target concentration is lower than 28.08 mol/m^3^, the high-concentration region increases with the target concentration. When the target concentrations are more than 28.08 mol/m^3^, the concentration distributions were unchanged.

The reason may be the limitation of the volume factor [47]. With the volume factor 0.1, the maximum volume of flow channels accounts for 10%, so it is difficulty to increase the optimised average concentration in the hydrogel continuously. 

### 3.3. Effect of Volume Factor on Optimisation Results

**Figure 11:**
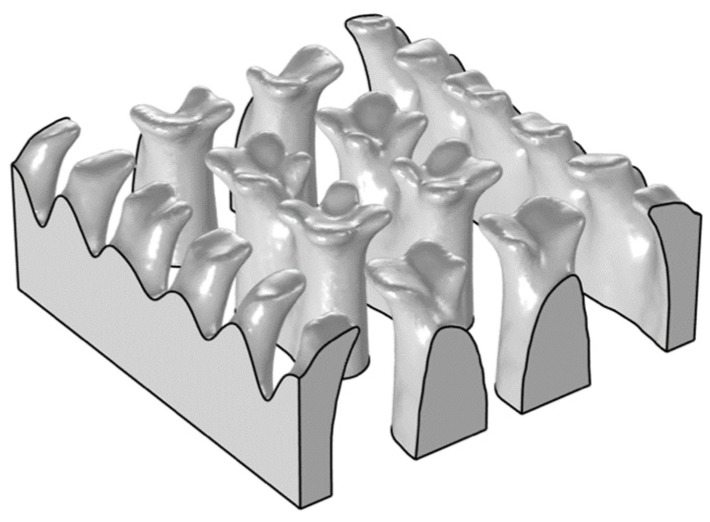
Flow channel structure at a volume factor of 0.2.

From Section 3.2, it is evident that the maximum adjustable average drug concentration in the hydrogel is 28.08 mol/m^3^ at a volume factor of 0.1. In order to meet a higher target concentration, the target concentration was set to 31 mol/m^3^, the volume factor was set to 0.2. The optimisation results showed that the average concentration in the hydrogel can reach the target concentration of 31 mol/m^3^. The structure of the flow channel is shown in Figure 11. The higher the proportion of flow channels in the hydrogel, the easier it is for the drugs to diffuse. So the increase in the volume factor leads to an increase in the maximum adjustable concentration in the hydrogel.

To obtain a relationship between the maximum adjustable concentration in the hydrogel and volume factors, the target concentration was set to be a saturated concentration of tetracycline hydrochloride of 41.59 mol/m^3^. The volume factors were set to 0.1, 0.2, 0.3, 0.4, and 0.5, respectively, the remaining parameters were unchanged for the structural optimisation.

**Figure 12:**
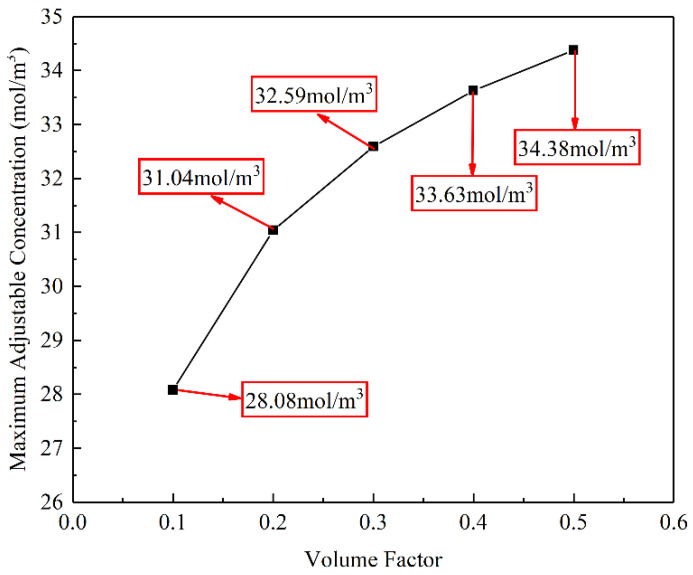
Relationship between the volume factor and the maximum adjustable concentration.

Figure 12 shows the relationship between the volume factor and maximum adjustable concentration. It is evident that the maximum adjustable concentrations in the hydrogel gradually increase with the volume factor. At a volume factor of 0.5, the maximum adjustable concentration in the hydrogel is increased to 34.38 mol/m^3^.

**Figure 13:**
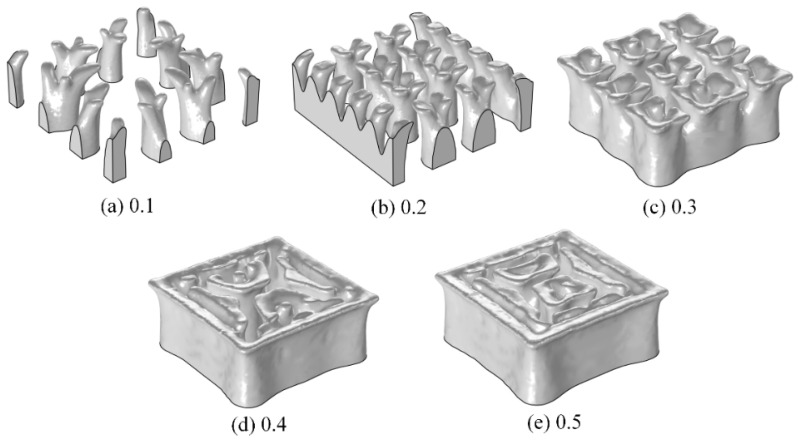
Flow channel structures with different volume factors.

Figure 13 shows the optimised structures of the flow channels under different volume factors. With an increase in the volume factor, the branches of the flow channels gradually disappear, and little flow channels are blended into a larger flow channel, which influences the strength of hydrogel CDDS. Considering the concentration and the strength at the same time, a volume factor of 0.2 is recommended, and the corresponding maximum adjustable concentration is 31.04 mol/m^3^. So that a reasonable adjustable range of the target concentration in hydrogel is 20.79 to 31.04 mol/m^3^.

### 3.4. Hydrogel CDDS In Vitro Experiment

**Figure 14:**
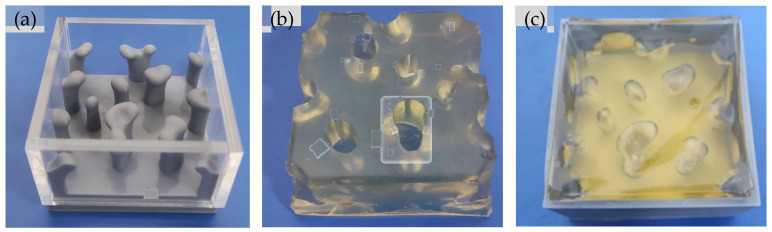
Photographs of the mould and sample used in in vitro drug release experiment: (**a**) Mould of the hydrogel flow channels; (**b**) Prepared topological hydrogel; (**c**) Hydrogel CDDS sample.

**Figure 15:**
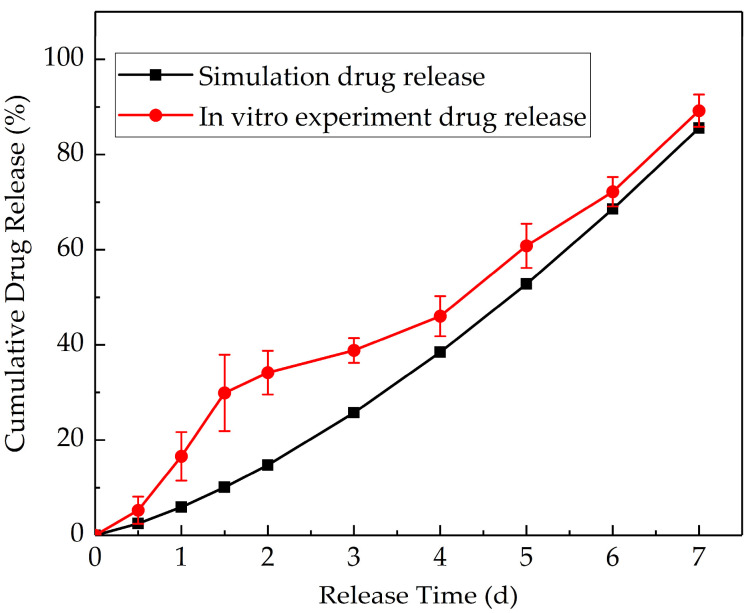
Comparison of in vitro drug release experimental and simulation results.

To verify the rationality of the optimal design of hydrogel topology structure, drug release experiments of the designed CDDS were conducted in vitro. The flow channels’ mould was prepared by 3D printing. The sample of the mould and the hydrogel CDDS are shown in Figure 14. The relationship between the cumulative drug release and release time is shown in Figure 15.

Figure 15 shows the simulation and experimental results of the cumulative drug release. The results show that the concentration of tetracycline hydrochloride already reached 135.4 μg/mL at the drug release 1.5 days, this value is higher than the MIC of tetracycline hydrochloride (89.2 μg/mL) [48]. Furthermore, the properties of tetracycline hydrochloride in the polyacrylamide/sodium alginate hydrogel are stable [49]. In the follow-up test, because the average drug concentration in the CDDS is constant, a stable concentration gradient is obtained, and the cumulative drug release curve basically tends to the zero-order release. The simulation curve of the cumulative drug release shows a similar trend to the experimental results, and thus, the rationality of the optimisation method and model is verified.

## 4. Conclusions

In this study, the topological optimisation method was utilised in the structural design of a hydrogel CDDS to achieve a parameter-based adjustment of the average concentration level of tetracycline hydrochloride in the CDDS. The results indicate that the average concentration level of tetracycline hydrochloride in hydrogel can be adjusted in the range of 20.79 to 31.04 mol/m^3^. Based on in vitro experiments, the CDDS can control drug release within 7 days, while the cumulative release curve tends to zero-order release. In order to the designed CDDS meets clinical requirements, the model parameters can be adjusted according to the drug type and dosage of specific clinical requirements. However, this study was carried out as a one-way drug release case; future studies will focus on improving the ability of the simulation model to conform to actual situations.

Traditional medical dressings make it difficult to meet the diverse medication needs of patients. This study provides a novel approach to address this challenge and provides a theoretical support for the precise delivery of drugs in the context of precision medicine.

## Data Availability

The data presented in this study are available on request from the corresponding author.
